# Ultrafiltration in Japanese critically ill patients with acute kidney injury on renal replacement therapy

**DOI:** 10.1186/s40560-021-00590-4

**Published:** 2021-12-20

**Authors:** Koichi Kitamura, Koichi Hayashi, Shigeki Fujitani, Raghavan Murugan, Toshihiko Suzuki

**Affiliations:** 1 Department of Nephrology, Endocrinology and Diabetes, Tokyo Bay Urayasu Ichikawa Medical Center, 3-4-32 Todaijima, Urayasu, Chiba 279-0001 Japan; 2 Department of Emergency and Critical Care Medicine, St. Marianna University School of Medicine, Kanagawa, Japan; 3 The Center for Critical Care Nephrology, Department of Critical Care Medicine, University of Pittsburgh School of Medicine, Pittsburgh, PA USA; 4 The Clinical Research, Investigation, and Systems Modeling of Acute Illness (CRISMA) Center, Department of Critical Care Medicine, University of Pittsburgh School of Medicine, Pittsburgh, PA USA

**Keywords:** Net ultrafiltration, Diuretics, Fluid overload, Renal replacement therapy

## Abstract

A recent worldwide survey indicates an international diversity in net ultrafiltration (UF^NET^) practices for the treatment of fluid overload in critically ill patients with acute kidney injury (AKI) requiring renal replacement therapy (RRT). The sub-analysis of the survey has demonstrated that maximum doses of furosemide used before determination of diuretic resistance are lower in Japan than those prescribed worldwide and UF^NET^ is lower but is initiated earlier. In contrast, the interval during which practitioners evaluate fluid balance is longer. The characterization of RRT in critically ill patients in Japan should unveil more appropriate approaches to the successful treatment of AKI.

**Letter to the editor**

## Introduction

Although renal replacement therapy (RRT) is an indispensable modality for the treatment of acute kidney injury (AKI), there have been no definitive guidelines for the appropriate management of fluid overload in critically ill patients with AKI. Recently, Murugan et al. [1] conducted a questionnaire-based worldwide survey and demonstrated wide variations of practice in net ultrafiltration (UF^NET^) prescription and practice. We had an opportunity to obtain the data reported by Japanese practitioners that constituted a part of the multinational results [1].

## Methods

We evaluated the UF^NET^ practice (timing of UF^NET^ initiation/UF^NET^ prescription) and the doses of diuretics in Japan. The results were compared with the multinational (not including Japan) survey.

## Results

**Fig. 1:**
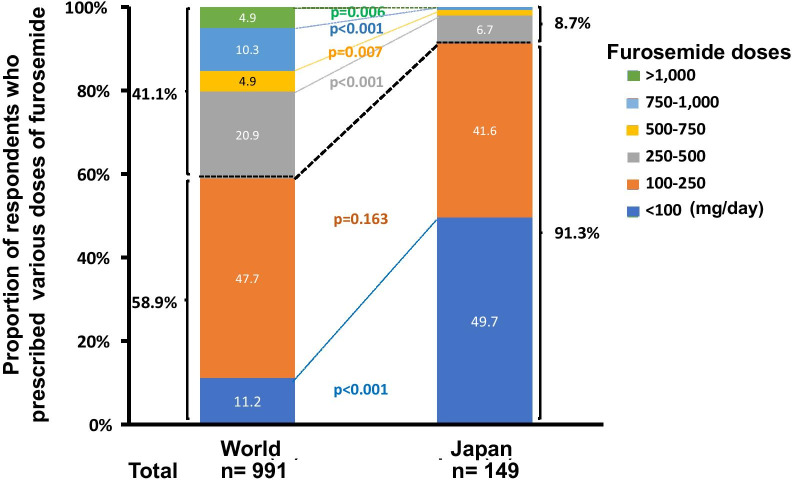
Maximum doses of furosemide prescribed by survey practitioners. Statistical analyses were performed using the Chi-square test or Fisher’s exact test, as appropriate. Evaluation between two cohort groups (world vs. Japan) was conducted using a two-proportion *z* test

We found a marked difference in the use of diuretics between these surveys. In the multinational survey, 41.1% of the practitioners used furosemide at a maximum dose of 250 mg/day or higher before determining diuretic resistance (Fig. 1). In contrast, 91.3% of Japanese doctors prescribed it at maximum doses lower than 250 mg/day.

Table 1 shows that 50.9% of the multinational respondents would start UF^NET^ after identifying persistent (≥ 12 h) oliguria/anuria; in Japan, only 26.6% of the practitioners commence UF^NET^ after persistent oliguria/anuria. Although hemodynamic status and cumulative fluid balance constitute two major determinants of the UF^NET^ prescription in both Japan and the world, 13.8% of Japanese practitioners pay priority attention to weight gain as a result of total fluid homeostasis.

**Table 1:** Comparison of parameters associated with UF^NET^ between world and Japan

	World (not including Japan)	Japan	*p* value
Q-1. Criteria used for UF^NET^ initiation, *N* (%)			
(a) Persistent oliguria/anuria (urine output < 0.5 mL/kg/h for ≥ 12 h)	477 (50.9)	38 (26.6)	< 0.001
(b) Severe hypoxemia (PaO_2_/FIO_2_ ratio < 150)	134 (14.3)	18 (12.6)	0.586
(c) Pulmonary edema with or without hypoxemia	162 (17.3)	32 (22.4)	0.138
(d) Cumulative fluid balance (> 1000 mL)	51 (5.4)	3 (2.1)	0.088
(e) Fluid overload > 10% of body weight	57 (6.1)	3 (2.1)	0.053
(f) Ongoing need for fluids in the presence of oliguria	57 (6.1)	49 (34.3)	< 0.001
Total	938	143	
Q-2. Criteria used for UF^NET^ prescription, *N* (%)			
(a) 24-h fluid balance	148 (14.8)	8 (5.3)	0.001
(b) Cumulative fluid balance	201 (20.1)	29 (19.1)	0.765
(c) Weight gain	48 (4.8)	21 (13.8)	< 0.001
(d) Radiographic features of fluid overload	21 (2.1)	10 (6.6)	0.002
(e) Hemodynamic status (HR, BP, CVP, PPV, dose of vasopressors)	552 (55.3)	82 (53.9)	0.763
(f) Volume of anticipated fluid use in the next 24 h	22 (2.2)	1 (0.7)	0.205
(g) Arterial lactate	7 (0.7)	1 (0.7)	0.953
Total	999	152	
Q-3. Method used to achieve UF^NET^ using CRRT, *N* (%)			
(a) By varying ultrafiltration rate only	536 (48.6)	77 (54.2)	0.203
(b) By varying replacement fluid rate only	60 (5.4)	20 (14.1)	< 0.001
(c) By varying both ultrafiltration and replacement fluid rate	508 (46.0)	45 (31.7)	0.001
Total	1104	142	
Q-4. How frequently did you check net fluid balance during CRRT? *N* (%)			
(a) ≤ Every 1 h	409 (35.8)	32 (21.9)	< 0.001
(b) ≤ Every 2 h	511 (44.8)	60 (41.1)	0.398
(c) ≤ Every 4 h	660 (57.8)	89 (61.0)	0.473
(d) ≤ Every 6 h	772 (67.7)	104 (71.2)	0.383
(e) ≤ Every 8 h	884 (77.5)	127 (87.0)	0.009
(f) ≤ Every 12 h	1015 (89.0)	136 (93.2)	0.121
(g) ≤ Every 24 h	1141 (100)	146 (100)	na

Statistical analyses were performed using the Chi-square test or Fisher’s exact test, as appropriate

*na* not applicable

In the worldwide survey, UF^NET^ was controlled by altering ultrafiltration rate or modulating both ultrafiltration and replacement fluid rate for hemodynamically unstable patients (Table 1). In Japan, however, fewer practitioners modified both parameters (31.7% vs. 46.0%). Finally, there observed was a marked variation in the frequency with which practitioners checked fluid balance during continuous RRT; hourly UF^NET^ evaluations were conducted by 35.8% of multinational but by only 21.9% of Japanese practitioners.

## Discussion

This sub-analysis unveiled that most of the Japanese doctors prescribed furosemide at maximum doses lower than 250 mg/day whereas the multinational survey [1] as well as the sub-analysis from Europe [2] showed the prescription of a maximum 250 mg/day or higher by 41.1–56.1% of physicians. Notably, in a study of acute heart failure management in Japan, the maximum dose of furosemide (≤ 200 mg/day) was less than half the dose used in the USA [3], which was expected to cause lower mortality [4]. In AKI, large doses of furosemide may cause ototoxicity [5] and, along with the prolonged infusion to delay dialysis, may be associated with a higher mortality [6].

Most practitioners (90.0%) across the world agree that early UF^NET^ is beneficial [1]. The present study suggests earlier implementation of UF^NET^ in Japan than in the world, possibly because Japanese practitioners have made early decision of diuretic resistance and recognition of weight gain resulting in identifying persistent oliguria/anuria in less than 12 h. It may fairly be presumed that early UF^NET^ initiation facilitates well-balanced fluid homeostasis and enables simultaneous administration of fluid volumes, including medications and nutrition [7]. Caveat is in order since there exists some controversy regarding the aggressive fluid management in critically ill patient [8, 9].

The UF^NET^ rate prescription is reported to be lower in Japan [40.0 mL/h] than in the worldwide survey [80.0 mL/h] [1]. There is an observational study suggesting J-shaped association between UF^NET^ rate and mortality in critically ill patients receiving RRT [10]; UF^NET^ rate between 1.01 and 1.75 mL/kg/h is associated with the lowest risk of death. Naorungroj et al. [11] have also shown that early UF^NET^ rate < 1.01 mL/kg/h is associated with decreased mortality when compared with early UF^NET^ rate > 1.75 mL/kg/h. Our survey and the original study by Murugan [1] evaluate UF^NET^ rate on the basis of mL/h, but if we assume the body weight of Japanese population as 57 kg (https://www.mhlw.go.jp/toukei/youran/indexyk_2_1.html), the UF^NET^ rate in Japanese population should be 0.7 mL/kg/h. Furthermore, there is a difference in the way of controlling UF^NET^ (altering ultrafiltration rate or modulating both ultrafiltration and replacement fluid rate) between the world-wide survey and Japan; fewer Japanese practitioners attempted to modify both parameters than those among the worldwide survey. This difference might be attributed to the smaller anthropometric characteristics of Japanese patients or relatively less requirement of replacement fluid exchange due to early introduction of ultrafiltration. The association between low UF^NET^ and mortality in Japan needs to be more thoroughly investigated.

Finally, this survey found that Japanese practitioners evaluated net fluid balance less frequently. The reason for this difference might be that constraints of staffing affect the timing of evaluation of UF^NET^ balance. This important issue requires urgent improvement.

There exist substantial worldwide or practitioner-dependent variations in UF^NET^ strategies for AKI patients. Under the current status, where the strategy for the RRT in critically ill patients is not highly organized yet, well-defined approaches to RRT, including evidence-based guidelines, are required to offer more favorable treatment to critically ill patients with AKI and consequently, to obtain more consistent results.

## Data Availability

Data sharing is not applicable to this article as no datasets were generated or analyzed during the current study.

## Abbreviations

AKI: Acute kidney injury
RRT: Renal replacement therapy
UF^NET^: Net ultrafiltration rate
